# Good News? Assessing Time-Trends in Fidelity to Responsible Reporting of Suicide Guidelines in the Canadian News Media from 2019-2023

**DOI:** 10.1192/j.eurpsy.2025.518

**Published:** 2025-08-26

**Authors:** R. Whitley, J. Careau

**Affiliations:** 1 Psychiatry, McGill University; 2 Psychiatry, Douglas Research Centre, Montreal, Canada

## Abstract

**Introduction:**

Research indicates that detailed and sensational media coverage of suicide can contribute to suicide mortality, known as the Werther Effect. Other research indicates that responsible reporting of suicide can promote hope and resilience, which can reduce suicidality, known as the Papageno effect. This has led to the development of responsible reporting of suicide guidelines. In Canada, these are contained in a booklet called *Mindset,* which was co-created by journalists and mental health experts. *Mindset* lists bullet-pointed recommendations for journalists, and has been widely disseminated to newsrooms, journalism schools and media professionals across the country, in tandem with educational activities aimed at journalists by suicide prevention experts.

**Objectives:**

The overall aim of this study is to assess fidelity to *Mindset* responsible reporting of suicide guidelines in the Canadian media over a multi-year period. A secondary objective is to assess whether fidelity to responsible reporting of suicide guidelines during the COVID-19 pandemic was significantly different to pre/post pandemic reporting of suicide.

**Methods:**

We collected articles from 47 different Canadian national and regional news sources mentioning the word *suicide* on a daily basis from April 1^st^ 2019 to March 31st 2023 (N=3,232). Each article was read and assessed for adherence to 12 key suicide reporting guidelines using a purposely-built coding sheet, derived from the *Mindset* guidelines. Trends in reporting were analyzed through time series analyses using a GLARMA model in R. software, including measuring for change during the COVID-19 pandemic.

**Results:**

Significant increases in putatively protective content were observed over the four years, including rises in the inclusion of help-seeking information and the quotation of experts. Similarly, there was a general decrease in content that is putatively harmful, for example significantly fewer articles in latter years gave a monocausal explanation of suicide, used sensational language or described in detail any suicide method. That said, fewer than one-third of articles in the final year included educational content about suicide, help-seeking information, or quotes from suicide experts. Reporting of suicide during the COVID-19 pandemic showed some positive improvements compared to pre-pandemic reporting, but these were not sustained post-pandemic.

**Conclusions:**

Fidelity to responsible reporting of suicide guidelines improved over the four-year period, especially recommendations concerning putatively helpful content. However there remains room for improvement regarding inclusion of putatively protective content such as including help-seeking information and educating the public about suicide. As such, further educational outreach to journalists is needed to encourage greater inclusion of protective content, while avoiding harmful content.

**Disclosure of Interest:**

None Declared

